# A dynamic meiotic SUN belt includes the zygotene-stage telomere bouquet and is disrupted in chromosome segregation mutants of maize (*Zea mays* L.)

**DOI:** 10.3389/fpls.2014.00314

**Published:** 2014-07-11

**Authors:** Shaun P. Murphy, Hardeep K. Gumber, Yunyun Mao, Hank W. Bass

**Affiliations:** ^1^Institute of Molecular Biophysics, Florida State UniversityTallahassee, FL, USA; ^2^Department of Biological Science, Florida State UniversityTallahassee, FL, USA

**Keywords:** telomere, SUN, nuclear envelope, bouquet, meiosis

## Abstract

The nuclear envelope (NE) plays an essential role in meiotic telomere behavior and links the cytoplasm and nucleoplasm during homologous chromosome pairing and recombination in many eukaryotic species. Resident NE proteins including SUN (Sad-1/UNC-84) and KASH (Klarsicht/ANC-1/Syne-homology) domain proteins are known to interact forming the Linker of Nucleoskeleton and Cytoskeleton (LINC) complex that connects chromatin to the cytoskeleton. To investigate the possible cross-kingdom conservation of SUN protein functions in plant meiosis, we immunolocalized maize SUN2 using 3D microscopy of pollen mother cells from maize (*Zea mays* L.), a large-genome plant model with a canonical NE zygotene-stage telomere bouquet. We detected SUN2 at the nuclear periphery and found that it exhibited a distinct belt-like structure that transitioned to a half-belt during the zygotene stage and back to a full belt during and beyond the pachytene stage. The zygotene-stage half-belt SUN structure was shown by 3D immuno-FISH to include the NE-associated telomere cluster that defines the bouquet stage and coincides with homologous chromosome synapsis. Microtubule and filamentous actin staining patterns did not show any obvious belt or a retracted-like structure other than a general enrichment of tubulin staining distributed widely around the nucleus and throughout the cytoplasm. Genetic disruption of the meiotic SUN belt staining patterns with three different meiosis-specific mutants, *desynaptic (dy1), asynaptic1 (as1)*, and *divergent spindle1 (dv1)* provides additional evidence for the role of the nuclear envelope in meiotic chromosome behavior. Taking into account all of the observations from this study, we propose that the maize SUN belt is directly or indirectly involved in meiotic telomere dynamics, chromosome synapsis, and possibly integration of signals and forces across the meiotic prophase nuclear envelope.

## Introduction

In recent years, investigations from many laboratories working on different species have revealed a conserved family of proteins that form a functional bridge between the nucleoplasm and the cytoplasm. These nuclear envelope (NE) bridge proteins, defined by having a Sad1p/UNC-84 homology (SUN) domain, are found in animals, yeast, and plants (Hiraoka and Dernburg, 2009; Starr, 2009; Starr and Fridolfsson, 2010; Zhou and Meier, 2013). The SUN proteins share certain structural characteristics, the most prominent of which is the presence of the conserved SUN domain itself (pfam07738: Sad1_UNC), typically located at or near the C-terminal portion of the protein. In addition to the SUN domain itself, most SUN domain proteins also contain one or more transmembrane domains and one or more coiled-coil domains. The SUN domain resides in the perinuclear space of the NE, and the N-terminal region alone is required for NE localization (Hodzic et al., 2004).

Genetic, biochemical, and cellular studies have revealed that these proteins reside and function in the inner nuclear membrane (INM) of the NE (Crisp et al., 2006). In the lumen of the NE, the SUN domains interact with other proteins that contain a Klarischt, Anc-1, and Syne Homology (KASH) domain (Luxton and Starr, 2014). These connections and interactions provide a structural chain of proteins capable of transducing forces from the cytoplasm to the nucleus or its contents. SUN proteins contribute to multiple different biological functions, including nuclear migration and anchorage within the cell, tethering of chromatin to the NE, and telomere dynamics required for proper meiotic chromosome segregation (reviewed by Starr and Fridolfsson, 2010).

One highly conserved function for meiotic SUN proteins in eukaryotes, is in meiosis, to tether and move telomeres during chromosome pairing and recombination (Tomita and Cooper, 2006, 2007; Ding et al., 2007; Schmitt et al., 2007; Fridkin et al., 2009; Hiraoka and Dernburg, 2009; Roberts et al., 2013). During meiotic prophase I, telomeres move to the NE, cluster into a bouquet arrangement, and finally disperse along the surface of the INM. In maize, meiotic telomere clustering has been demonstrated to occur *de novo* on the NE during meiotic prophase I, and the temporal patterns are nearly identical to those in mammals (Scherthan et al., 1996; Bass et al., 1997). Interestingly, genetic disruption of the *SUN1* gene in mice leads to defects in meiotic telomere-NE association, pairing, synapsis, and recombination, a phenotype remarkably similar to those of two maize synapsis-deficient mutants, *desynaptic* (*dy*) and *desynaptic1* (*dsy1*) (Bass, 2003; Ding et al., 2007).

The SUN proteins in plant species are also encoded by two small gene families, one encoding the animal-like C-terminal SUN proteins, and the other encoding the plant-prevalent mid-SUN proteins (Murphy et al., 2010). The functions and mechanisms of plant SUN-domain proteins are likely, therefore, to be varied and essential, as indicated by several recent studies and reviews (Graumann et al., 2010, 2013; Murphy et al., 2010; Oda and Fukuda, 2011; Murphy and Bass, 2012; Zhou et al., 2012, 2014; Zhou and Meier, 2013; Evans et al., 2014). A meiosis-specific SUN-containing LINC-type complex is presumed to exist in plants, given the conservation SUNs. A plant meiotic LINC-type complex would likely mediate meiotic telomere movements and nuclear dynamics associated with early and middle prophase I (Murphy and Bass, 2012). In this study, we used new peptide antibodies for detection of C-terminal maize SUN proteins to examine the question of meiotic SUN function in maize. We discovered a novel structure, herein called a meiotic SUN belt, and found that it exhibits stage-specific changes in morphology, contains the telomere bouquet at zygotene, and is susceptible to genetic disruption by mutations historically defined as meiosis-specific chromosome segregation mutants.

## Materials and methods

### Plant materials

Inbred lines of maize (*Zea mays* L., 2*n* = 2X = 20) were grown, and pre-emerged meiotic tassels were harvested throughout the year from either field-grown plants or from plants grown under long-day (16 h. light) conditions in the greenhouse at the Mission Road Research Facility (Tallahassee, FL). Maize lines used were either wild-type inbred lines (A344, KYS), or meiotic mutants, *desynaptic* (*dy*), *asynaptic1* (*as1*), or *divergent spindle1* (*dv1*), obtained from the maize genetics cooperation stock center (http://maizecoop.cropsci.uiuc.edu/).

### Polyclonal antisera production

Two regions of the ZmSUN2 protein sequence were selected for production of rabbit polyclonal peptide antisera. The synthetic peptides used were designated SUN2-NEPsp1 [Ac-LEDKRLALERQLT(C)-amide, with “(C)” indicating a residue added for synthesis] and SUN2-NEPsp2 [Ac-(C)LEHVSKDVAYDRS-amide]. These peptides were combined for immunization and the resulting antisera were designated anti-SUN2-NEPsp2. The antiserum was affinity-purified against both peptides, and the purified serum was designated as anti-SUN2-NPAP. Peptide synthesis, antibody production, and affinity purification against the synthetic peptides were carried out by New England Peptide (New England Peptide, Gardiner MA, USA).

### Western blotting

Total maize protein extracts were obtained as previously described (Murphy et al., 2010) with several modifications. Briefly, 2 g of plant tissue from maize inbred line A344 was harvested, flash-frozen in liquid nitrogen, and stored in 50 mL sterile polypropylene tubes at −80°C. Frozen tissue was ground into a powder in liquid nitrogen and transferred to a homogenizer containing 6 mL of ice-cold 1× protein extraction buffer [50 mM Tris-HCl (8.0), 1 mm EDTA-NaOH (8.0), 10% w:v sucrose, 100 mM dithiothreitol, and 1× protease inhibitor complex] [4-(2-aminoethyl) benzenesulfonyl fluoride, bestatin, pepstatin-A, E-64, leupeptin, and 1,10-phenanthroline, Sigma Aldrich], and homogenized for 3 min on ice. The homogenate was centrifuged at 12,000× g for 20 min at 4°C, and the supernatant was recovered and used immediately for immunoblotting or stored at −80°C.

For western analyses, protein extracts were fractionated on 10% SDS acrylamide gels and transferred to a polyvinylidene fluoride (PVDF) transfer membrane (PALL life sciences, Port Washington, NY) in a Bio-Rad Mini-PROTEAN 3 Cell as described (Murphy et al., 2010). After transfer and blocking, the membranes were incubated with α-SUN2 NPAP diluted 1:20,000 with PBS-T at room temperature for 1 h. After four 15-min washes in PBS-T buffer at room temperature, the membranes were incubated with a 1:10,000 dilution (in PBS-T buffer) of anti-rabbit IgG horseradish peroxidase–linked antibody1 (Santa Cruz Biotechnology, Santa Cruz, CA) for 1 h at room temperature, then subjected to 4 × 15 min washes in PBS-T buffer at room temperature. The immune complexes were visualized with a chemiluminescent reaction kit for 0.5–3 min at room temperature (Millipore, Immobilon detection kit, WBKL50100, Billerica, MA).

### Fixation and preparation of maize cells

Cells were prepared essentially as described previously (Murphy et al., 2010; Murphy and Bass, 2012) with slight modifications. Meiosis stage anthers were microdissected and fixed in 1× Meiocyte Buffer A (Howe et al., 2013) supplemented with 1% paraformaldehyde and 0.05% Tween-20 for 1 h at room temperature. Anthers were then rinsed in 1× Buffer A alone on a rotary shaker for 30 min at room temperature, and finally stored for several weeks at 4°C in 1× Buffer A. Variations on this method are described below.

**Figure 1:**
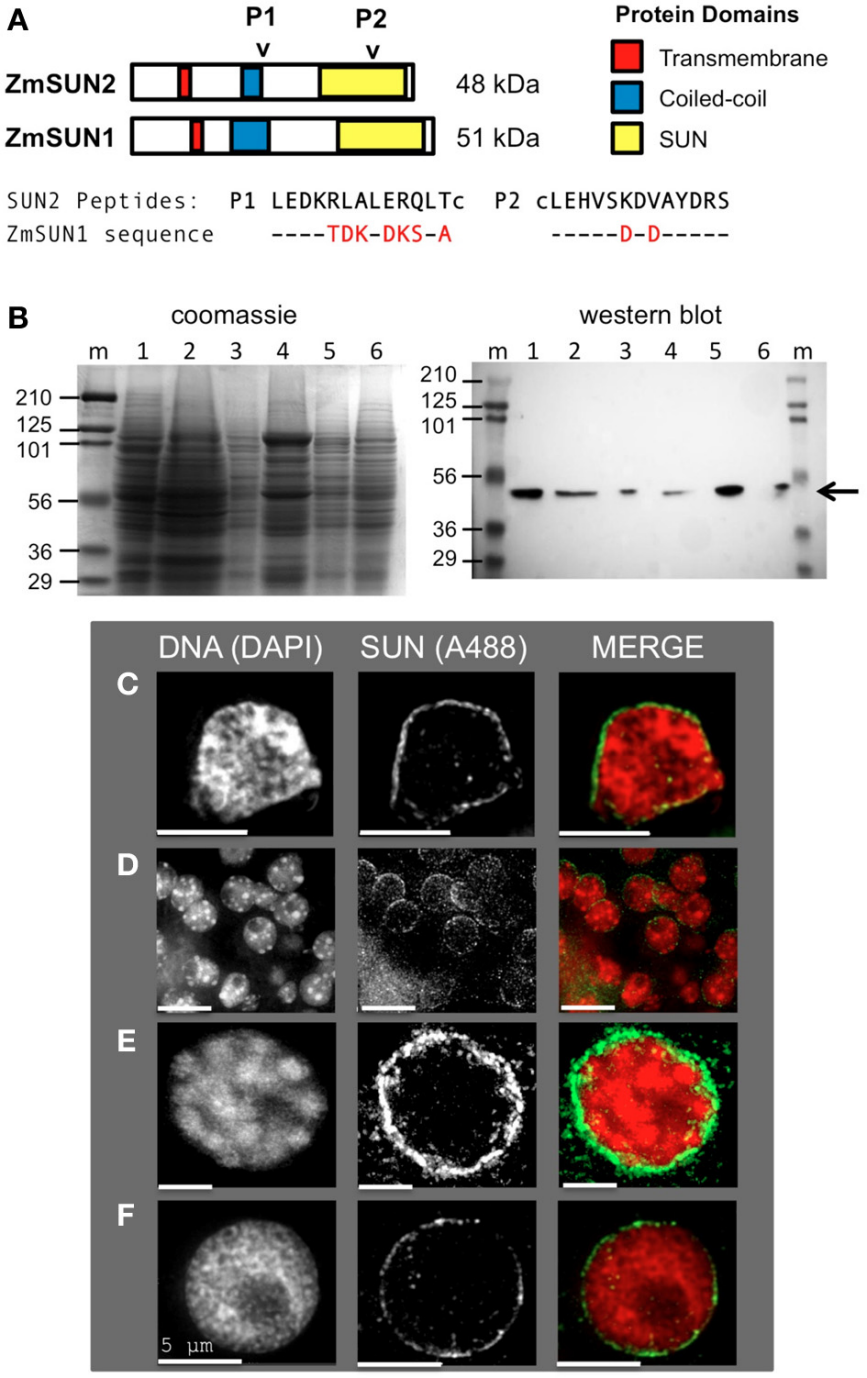
**Expression and localization of the maize CCSD class of SUN proteins in somatic tissues. (A)** Generation of two synthetic peptides (SUN2-P1 and SUN2-P2, arrowheads) for immunolocalization of the endogenous CCSD class of maize SUN proteins ZmSUN2, and ZmSUN1 is shown. Peptide alignments for SUN1 and SUN2 are indicated, with divergent residues highlighted in red. Putative protein domains based on prediction algorithms are indicated (colored boxes). Lowercase cysteine (“c”) residues were synthetically added for affinity purification. **(B)** Immunological detection of endogenous SUN proteins in maize somatic cells from a variety of maize organs; Lanes (1) meiotic tassel primordia, (2) emerged tassel, (3) earshoot, (4) leaf, (5) husk, (6) silk, (m) marker lanes for protein size (kDa, indicated at left) standards. Total proteins from equal fresh-weight plant material were fractionated on duplicate gels, one for SDS-PAGE gel followed by Coomassie staining (left panel) and the other for western analysis (right panel). The arrow indicates a single detectable species smaller than 56 kDa. **(C–F)** Protein immunolocalization using antisera indicated from **(C)** isolated earshoot nuclei, **(D)** somatic nuclei from cryo-sectioned whole anthers, **(E)** somatic prophase nuclei from isolated meiotic anthers and **(F)** pre-meiotic interphase nucleus. DAPI (total DNA, red) and A488 (SUN, green) are indicated in the merged 2D projections of the 3D data sets. Scale bars = 5 μm.

Somatic ear-shoot nuclei were isolated as previously described (Fincher et al., 2013). For the tissue section shown in Figure 1D, fixed meiotic anthers were sectioned and mounted on the slide without acrylamide;: tissues were harvested and fixed in 1× meiocyte Buffer-A supplemented with 0.05% tween-20 and 1% paraformaldehyde. Intact anthers in early meiotic prophase were submerged in a disposable vinyl specimen mold (10 × 10 × 5 mm) in 2 mL of Tissue-Tek optimal cutting temperature (O.C.T., 10.24% polyvinyl alcohol, 4.26% polyethylene glycol, and 85% non-reactive resins) compound, and incubated at −80°C for 30 min. The specimen molds were removed from the −80°C freezer and immediately sectioned at 50 μm on a microtome set to −20°C, placed on poly-L-lysine coated glass slide, and incubated at −20°C prior to protein immunolocalization.

### SUN immunocytochemistry

Fixed cells were embedded in polyacrylamide, followed by a 1-h room-temperature treatment in permeabilization buffer (1% Triton X-100, 1 mM EDTA-NaOH, and 1% BSA in 1× PBS). The acrylamide pads on the slides were then incubated in blocking buffer (3% BSA, 5% normal sheep serum, 1 mM EDTA-NaOH, 0.1% Tween-20, and 1 mM DTT in 1× PBS) at room temperature for 2 h and then incubated with the primary antibody (α-SUN2 /1 NEP-AP, at a dilution of 1:250 in blocking buffer) overnight at room temperature. After eight consecutive 30 min washes at room temperature with 1× PBS, cells were incubated with a FITC-conjugated goat anti-rabbit IgG (at 1:1000 in blocking buffer) overnight at 4°C then given four 15-min washes with 1× PBS at room temperature. Chromosomes were then stained with 3 μg/mL DAPI (4′, 6-diamidino-2-phenylindole) in 1X PBS for 30 min at room temperature, rinsed three times with 1× PBS, mounted in VectaShield anti-fading solution.

### Cytoskeletal staining

For microtubule staining, meiosis-staged anthers (wild-type inbred line A344) were fixed in 8% paraformaldehyde plus 1× PHEMS buffer (60 mM PIPES, 25 mM Hepes, 10 mM EGTA, 2 mM MgCl_2_, 0.32 M sorbitol, pH 6.8) for 2 h at room temperature essentially as described (Chan and Cande, 1998). Anthers were subsequently washed in 1× PHEMS and stored at 4°C or microdissected and embedded in polyacrylamide (Roberts et al., 2013) followed by a 1 h room-temperature treatment in permeabilization buffer (0.01% Triton X-100, 1 mM EDTA-NaOH, and 1% BSA in 1× PBS). The acrylamide pads on the slides were then incubated with 1% BSA in 1× PBS at room temperature for 1 h and then incubated with the primary antibody (monoclonal TUB 2.1, Cat. No T5201, Sigma-Aldrich) at a dilution of 1:300 in 0.5% BSA overnight at room temperature. Secondary antibody (goat anti mouse—Alexa594) was used at 1:1000 for 3–16 h, followed by counterstaining with DAPI and mounting in VectaShield for imaging.

For microfilament staining, anthers were fixed with formaldehyde plus glutaraldehyde and actin microfilaments were stained with using Rhodamine Phalloidin (Life Technologies). Fixed anthers were microdissected, washed in PBS, and permeabilized in 1% Triton X-100 /PBS. 1% BSA/PBS was used to block the non-specific binding. Cells were stained in 3.3 μM Rhodamine Phalloidin for 1 h at room temperature, washed and counterstained with DAPI (0.1 μg/m in PBS), and mounted in VectaShield for imaging.

### SUN telomere double staining by immuno-FISH

Immuno-FISH analysis of the maize CCSD class of SUN proteins with telomeres was carried out using fixed meiotic nuclei (wild-type inbred line A344). Immunolocalization was performed as described above except that the immune complexes were detected with a rhodamine conjugated goat anti-rabbit IgG (at 1:1000 in blocking buffer), followed by a second fixation in meiocyte Buffer A supplemented with 4% paraformaldehyde at room temperature. Nuclei were washed for 30 min at room temperature in 1× PBS, and subjected to telomere FISH using a fluorescein-conjugated telomere-specific oligonucleotide probe, MTLF at 0.13 μM. Post-hybridization washes, DAPI staining, and mounting were done as previously described (Bass et al., 1997).

### 3D deconvolution microscopy and image processing

Multi-wavelength 3D deconvolution microscopy was used to image and visualize stained maize cells as previously detailed (Bass et al., 1997, 2014; Murphy and Bass, 2012). 3D model volume-rendered images were produced from the gray-scale datasets using the SotfWorx programs (DeltaVision, Applied Precision/GE Healthcare).

## Results

### Expression and localization of the maize C-terminal class of SUN proteins in somatic cells

Our previous findings suggested that the maize SUN protein gene family is expressed in somatic and meiosis-enriched tissues (Murphy et al., 2010). In order to examine the meiotic localization patterns in more detail, we developed antisera using synthetic peptides encoded by the C-terminal class of SUN protein genes *ZmSUN2* and *ZmSUN1*, as summarized in Figure 1A. The first peptide produced (Peptide 1, Figure 1A) is likely to detect the predicted coiled-coil region of ZmSUN2. This portion of the protein sequence is quite divergent compared to that region of ZmSUN1. The second synthetic peptide (Peptide 2, Figure 1A) would likely cross-react with the SUN domain of both ZmSUN2 and ZmSUN1. Western blot analysis using these affinity-purified anti-sera revealed a single species of ~50 kD in multiple maize tissues, including meiotic tassel primordia, emerged tassel, earshoot, leaf, husk, and silk (Figure 1B). The predicted molecular weights of SUN1 and SUN2 are 51 and 48 kDa (Figure 1B). The band detected (Figure 1B, arrow) most likely reflects specific detection of SUN2, but the possibility of detection of SUN1 as a co-migrating band cannot be entirely excluded.

We examined the sub-cellular localization of the detected proteins in fixed cells via indirect immunocytological staining. We found that the anti-SUN sera stained cells primarily around the nuclear periphery (Figures 1C–F). This pattern was seen in multiple cases, as shown for isolated earshoot nuclei (Figure 1C), in somatic cells from meiosis-stage anthers, (Figure 1D) and isolated nuclei at meiotic prophase (Figure 1E) and interphase (Figure 1F). The nuclear periphery staining patterns confirm the prediction that the C-terminal SUN proteins are resident NE proteins found in most cell types.

### The maize meiotic SUN belt, a novel structure around male meiotic prophase nuclei

**Figure 2:**
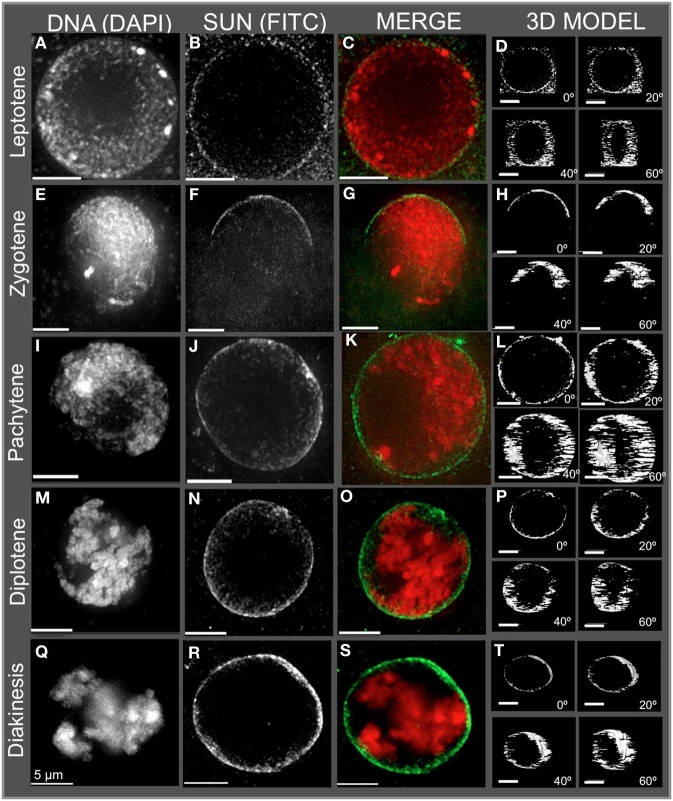
**Immunolocalization of the maize CCSD class of SUN proteins in meiotic cell nuclei**. Protein immunolocalization using reagents that detect endogenous SUN2 (and likely SUN1) proteins were used to stain **(A–C)** leptotene, **(E–G)** zygotene, **(I–K)** pachytene, **(M–O)** diplotene, and **(Q–S)** diakinesis stage meiotic nuclei from maize. DAPI (total DNA, red) and FITC (SUN, green) are indicated in the merged 2D projections of the 3D data sets. 3D models are presented in **(D,H,L,P,T)** and correspond to the meiotic nuclei for the indicated to the left. Rotation about the Y axis clearly shows the location of the meiotic SUN belt. 2D projections of the entire 3D data sets are presented. Scale bars = 5 μm.

We next examined localization in of these SUN proteins during meiotic prophase, where they are expected to carry out specialized functions associated with telomere clustering and nuclear movements during meiotic prophase. For this, we used the male meiotic prophase cells of maize, building on prior 3D molecular cytological plant meiosis studies (Bass et al., 1997, 2000, 2003; Murphy and Bass, 2012; Howe et al., 2013). On the basis of prior studies of meiotic prophase in fission yeast, budding yeast, and mouse (Chikashige et al., 2006; Conrad et al., 2007; Ding et al., 2007), we expected that our sera would stain punctate foci on the NE that reflected the position of attached telomeres. We detected instead a striking novel meiotic nuclear structure, termed here a meiotic SUN belt, characterized by SUN staining in a discrete belt-like pattern around the nucleus, as summarized in Figure 2.

The meiotic SUN belt is seen in leptotene (Figure 2A) as a lightly stained ring, and at zygotene as a partial ring or half-belt (Figures 2E–H), followed by return to a full belt at pachytene and late prophase (Figures 2I–T). To more clearly visualize this novel structure, we converted the SUN staining patterns into polygons and display them as projections of solid models viewed from different angles, rotated 0, 20, 40, and 60° about the Y axis (Figure 2. 3D model). The model projection clearly shows the half-belt nature of the zygotene (Figure 2H). Intriguingly, the meiotic SUN belt staining appeared to be limited to the central Z-sections, perpendicular to the plane of imaging. For comparison, we imaged fluorescent dye-coated microsphere beads, and found the staining pattern to be different from the maize meiotic SUN belt-type pattern, demonstrating that the belt was not an imaging artifact of mid-nuclear optical sectioning (Murphy and Bass, data not shown). Movies showing step-through sections and rotating projections of representative 3D image datasets are provided in Supplemental Table 1.

In summary, the meiotic SUN belt was found to display the following features: (1) it forms a discrete band around the nucleus located in a central medial arrangement, parallel to the plane of the slide, (2) it rearranges into a partial or a half-belt-like structure during zygotene, (3) it is restored to a more complete belt later in pachytene (4) and finally, it persists through diplotene and thus is present during entire length of meiotic prophase. These summary observations presented here are based on visual inspection (*n* > 500), and 3D data collection (*n* > 75) of maize meiocyte nuclei, with at least 25 3D images collected from early, middle, or late prophase.

### The telomere bouquet is located within the meiotic SUN belt

**Figure 3:**
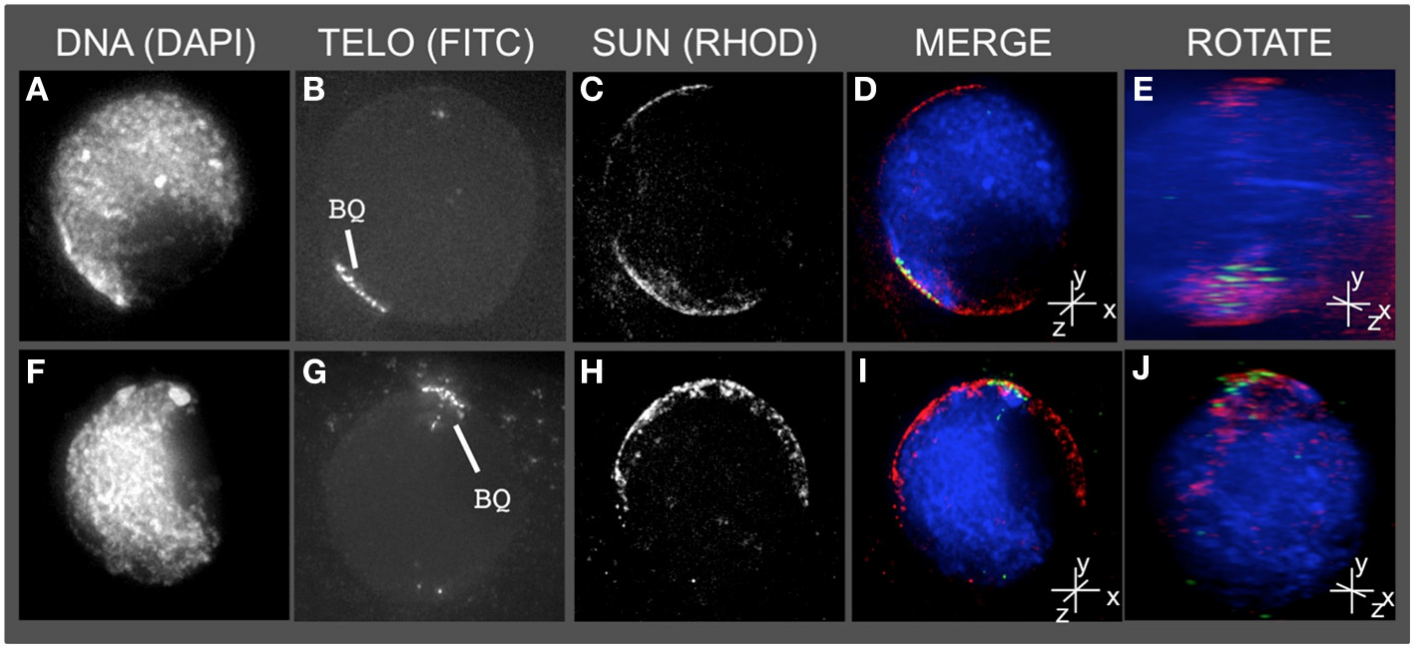
**The telomere cluster bouquet colocalizes with the SUN belt at zygotene**. Protein immunolocalization followed by 3D telomere FISH was used to analyze the location of meiotic telomeres relative to the distribution of SUN proteins in meiotic anthers harvested from maize inbred line A344+/+. 2D projections of the entire 3D data sets are presented for two different bouquet-stage meiotic nuclei. DAPI **(A,F)** (blue, DNA), FITC **(B,G)** (A488, green, telomere FISH signals), TRITC **(C,H)** (A546, red, SUN localization) are indicated in the merged projections **(D,I)**. Rotation about the y axes **(E,J)** clearly shows the location of the telomeres relative to the distribution of SUN proteins at the nuclear envelope. Scale bars = 5 μm.

Having detected this novel SUN structure, we tested the obvious prediction of colocalization of the bouquet telomere cluster on the NE and the SUN belt. Conservation of telomere-SUN-NE links in meiosis is well documented for many non-plant species (Hiraoka and Dernburg, 2009; Starr and Fridolfsson, 2010). Immuno-FISH was used to co-stain cells for SUN2 and telomeres. We found that the telomere FISH signals in the bouquet-stage nuclei were located within meiotic SUN belt proteins, as summarized for two representative nuclei in Figure 3. The 3D images clearly show that the telomere cluster (BQ, Figures 3B,G) overlaps with part of the meiotic SUN belt (color overlay, Figures 3D,E,I,J). The summary observations presented here are based on visual inspection (*n* > 50), and 3D data collection and analysis (*n* ~20) of maize meiotic nuclei at each meiotic prophase I stage. At subsequent stages in prophase I, when the telomeres no longer show NE association, the nuclear SUN belt persists (data not shown).

### Tubulin and actin staining do not localize to a conspicuous band around the nucleus

**Figure 4:**
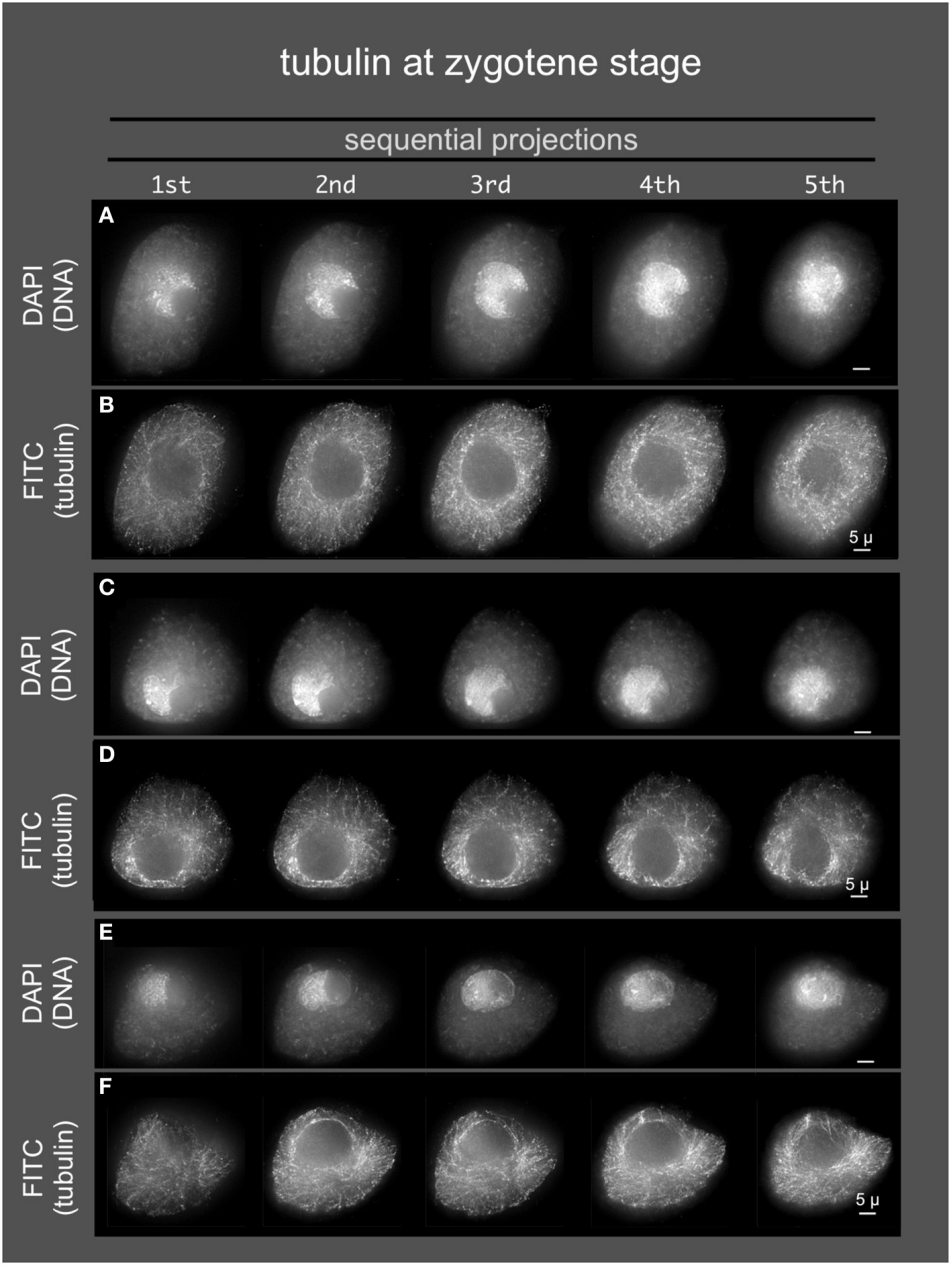
**Microtubules in early meiotic prophase (zygotene stage)**. Anthers from maize inbred line A344 were fixed and dissected meiotic cells were embedded in acrylamide gels for immunostaining using TUB2.1 antisera and, and counterstaining with DAPI. Three cells **(A,B, C,D,** and **E,F)** are shown as sequential projections from a series of partial projections (1/5 of the Z-sections each) made using the maximum intensity method.

**Figure 5:**
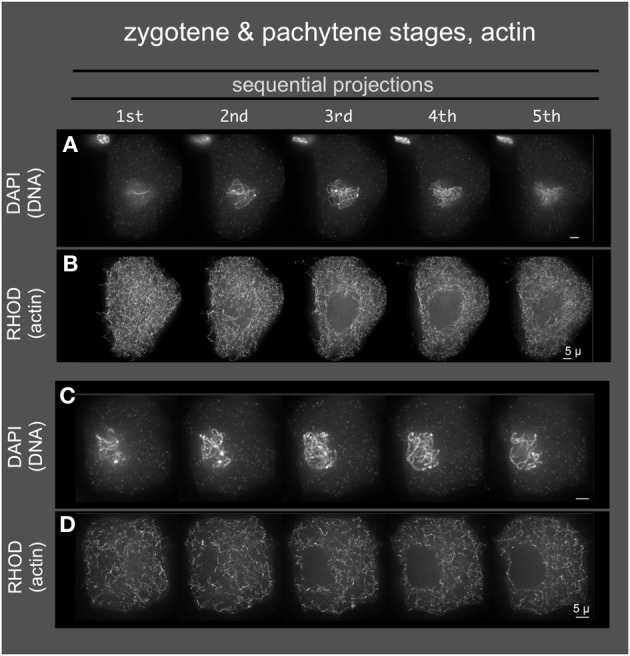
**Microfilaments in middle meiotic prophase (pachytene stage)**. Anthers from maize inbred line A344 were fixed and dissected meiotic cells were embedded in acrylamide gels for staining with Rhodamine Phalloidin, and counterstaining with DAPI. 3D images were collected, deconvolved, and image data was displayed as described in Figure 4. Two cells **(A,B**, and **C,D)** are shown.

Given the role of the cytoskeleton in meiosis, we examined the localization of microtubules and microfilaments as shown in Figures 4, 5, respectively. Previous studies describe the occurrence of a ring of perinuclear tubulin in some preparations of some plant meiotic cells (Shamina, 2005). Despite repeated efforts, we were unable to develop fixation and staining conditions that allowed for double-labeling of either tubulin or actin and SUN2. However, 3D tubulin staining (Figure 5) of zygotene-stage nuclei showed the typical pattern of diffuse microtubules with a slight distributed enrichment around the nucleus as a whole. We did not observe a distinct belt or band-like staining pattern. Similarly for actin staining, we observed distributed cytoplasmic staining (Figure 5), but no cases of belt or band-like structures that resembled the SUN2 patterns (Figure 2).

### Genetic disruption reveals a central role for the SUN belt in meiotic prophase

Previously characterized meiotic mutants of maize were used to test the idea that the meiotic SUN belt has an essential function in meiotic prophase, and would therefore be subjected to genetic disruption. For this we examined three different classical meiosis-specific mutants of maize that we deemed highly relevant to this study: *desynaptic, dy* (Nelson and Clary, 1952), *asynaptic1, as1* (Beadle and McClintock, 1928), and *divergent spindle1, dv1* (Clark, 1940).

The homozygous *dy* (*dy/dy*) mutation causes chromosome desynapsis at meiotic prophase, and has been shown to exhibit telomere-associated defects, NE distortion, synaptic irregularities, crossover control defects, chromosome bridges, and micronuclei, collectively resulting in aneuploidy and a semi-sterile phenotype (Nelson and Clary, 1952; Maguire et al., 1991; Ji et al., 1999; Bass et al., 2003; Murphy and Bass, 2012). The *dy* mutation was also recently mapped and a linked SUN protein gene (*ZmSUN3*, a PM3/mid-SUN type) was identified as a candidate gene (Murphy and Bass, 2012). The homozygous *as1* mutation (*as1/as1*) results in asynapsis at meiotic prophase, resulting in sterility (Beadle and McClintock, 1928). The homozygous *dv1* (*dv1/dv1*) mutation was selected for its known meiosis-specific contribution to both microtubule organizational defects and chromosomal segregation (Clark, 1940; Staiger and Cande, 1990; Shamina et al., 2000). The identity of the genes mutated in *as1* or *dv1* remains unknown.

**Figure 6:**
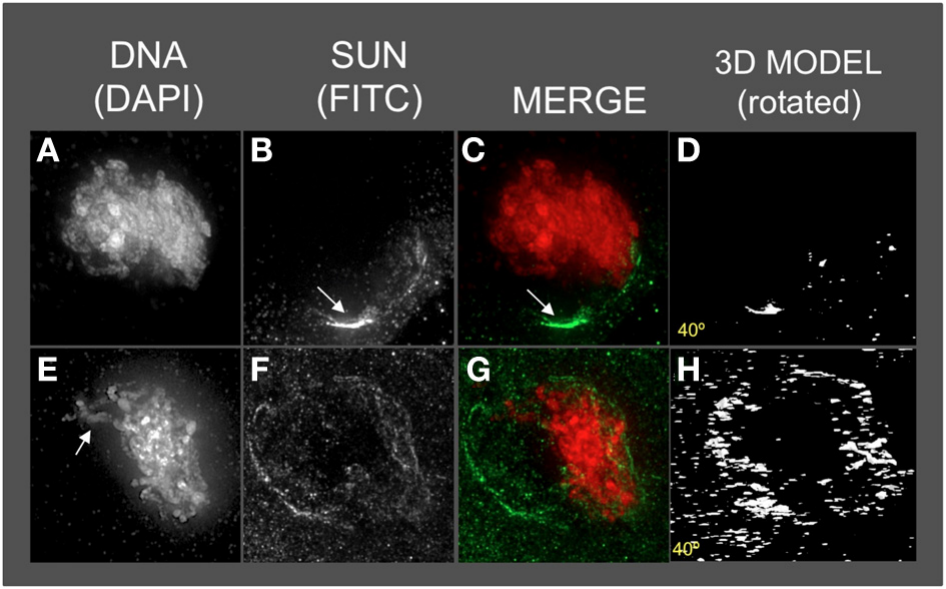
**The maize SUN2 in *dy1/dy1* meiotic cell nuclei**. Two representative nuclei **(A–D**, **E–H)** from cells fixed and stained as described in the legend for Figure 2. DAPI (total DNA, red) and FITC (SUN, green) images are shown along with projections/3D models of the FITC images viewed as rotations about the Y axis **(D,H)**. A bright patch of SUN staining (arrow, panels **B,C**) near the nucleolus (arrow in **B**) and a thick pachytene-like fiber (arrow in **E**) is indicated. Scale bars = 5 μm.

**Figure 7:**
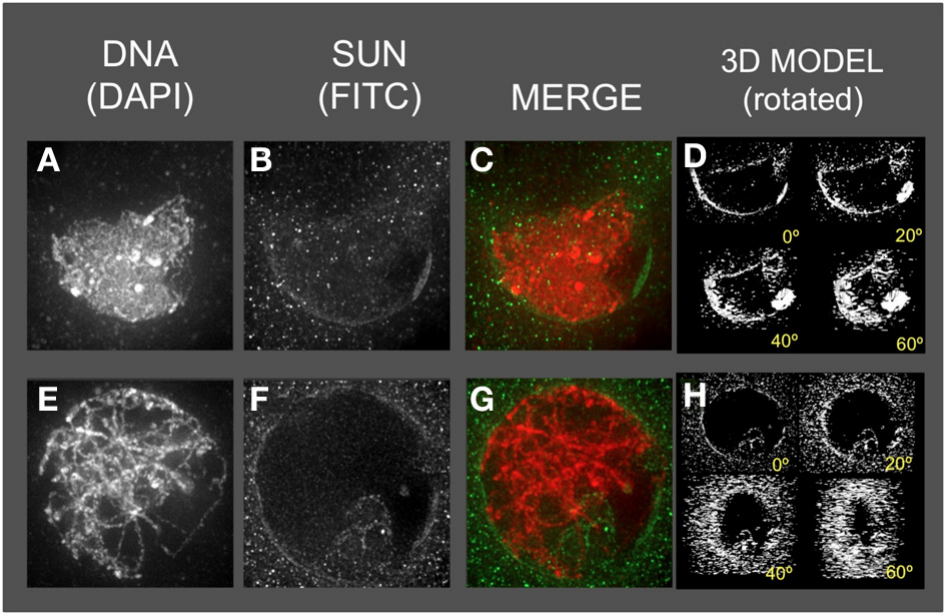
**The maize SUN2 in *as1/as1* meiotic cell nuclei**. Two representative nuclei **(A–D**, **E–H)** from cells fixed and stained as described in the legend for Figure 2. DAPI (total DNA, red) and FITC (SUN, green) images are shown along with projections/3D models of the FITC images viewed as rotations about the Y axis **(D,H)**.

**Figure 8:**
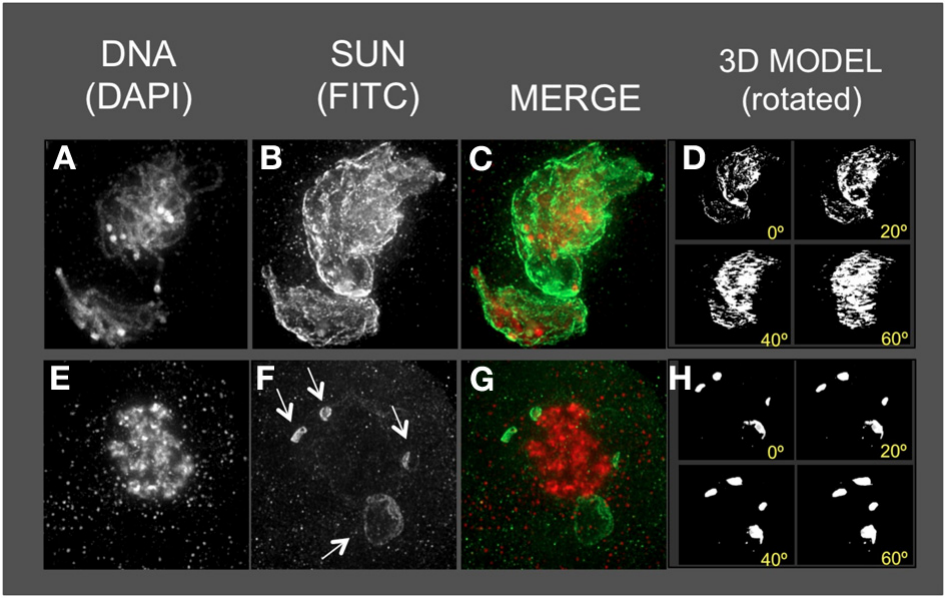
**The maize SUN2 in *dv1/dv1* meiotic cell nuclei**. Two representative nuclei **(A–D**, **E–H)** from cells fixed and stained as described in the legend for Figure 2. DAPI (total DNA, red) and FITC (SUN, green) images are shown along with projections/3D models of the FITC images viewed as rotations about the Y axis **(D,H)**. Aberrant SUN localization structures (arrows in **F**) are indicated.

**Figure 9:**
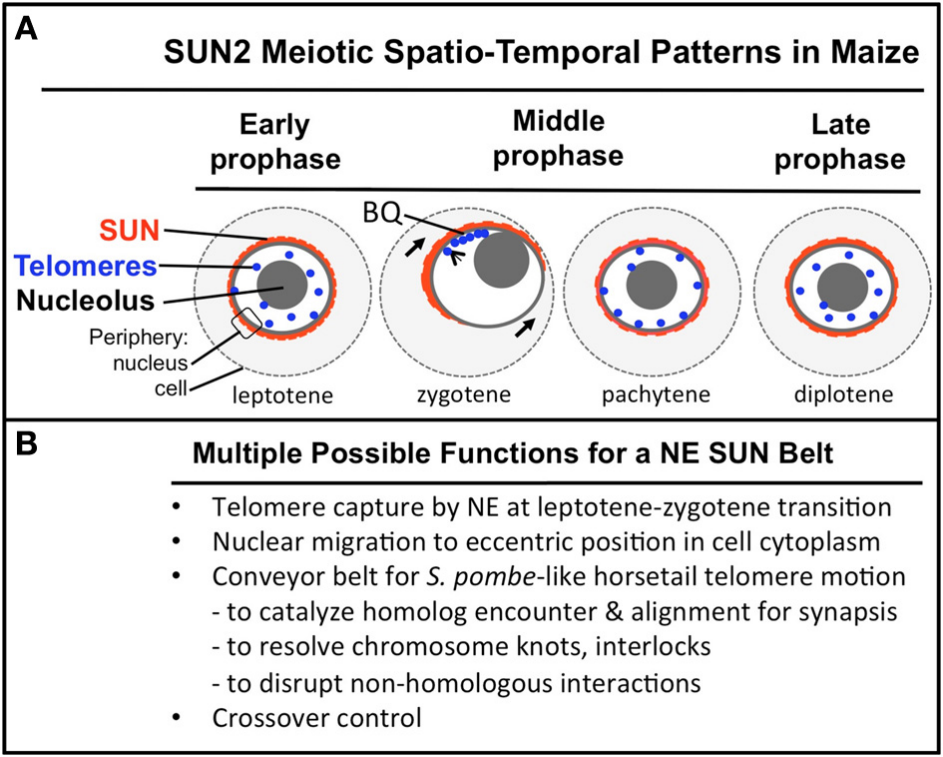
**A summary diagram of the meiotic SUN belt spatio-temporal staining patterns and possible functions**. A summary diagram **(A)** shows the relative distribution of SUN2 staining (red), telomeres (blue dots), nucleolus (gray circle), and the peripheries of the NE and cell. Stages of prophase are indicated at the bottom of each cell. The telomere bouquet (BQ) is indicted in zygotene as being localized within the partially retracted or polarized SUN belt signals. **(B)** Conceivable functions for the maize meiotic SUN belt are listed.

Mutant male meiocytes were harvested, fixed, and stained using the anti-SUN2 sera. The staging of meiosis is based on the cytological appearance of the chromosome fibers and their intra-nuclear arrangements. As a result, precise staging in meiotic mutants can be problematic and the mutant stages are more generally classified as early, middle, or late prophase to indicate their approximate developmental stage. Figures 6–9 show that each of the mutants resulted in altered SUN staining pattern and nuclear morphology. Phenotypes and SUN belt alterations ranged in severity from mild for *as1* to extreme for *dv1*.

Most *dy1* nuclei at early or middle prophase (Figures 6A–D) showed abnormal SUN distribution patterns, unlike those of the wild type nuclei with belts or a retracted half-belt structure. Instead, the SUN staining in *dy1* showed concentrated patches along a distorted, non-spherical nuclear periphery. A middle-prophase nucleus (Figures 6E–H) with pachytene-like fibers (arrow, Figure 6E) showed a less severe disruption with irregular and discontinuous SUN staining, punctate foci at the nuclear periphery, and some signals both inside the nucleus and in the cytoplasm (Figure 6F).

The *as1* nuclei (Figure 7) showed less severe disruption of the SUN distribution patterns. The SUN belt does appear to adopt the half-belt configuration, but with excessive staining in the cytoplasm compared to wild type. In addition, the SUN staining sometimes appeared to localize to regions of possible NE invaginations (Figure 7F, bottom of the nucleus).

The *dv1* nuclei showed the most extreme and remarkable alteration of SUN staining patterns, as seen for example at middle (Figures 8A–D) or late (Figures 8E–H) prophase. The nucleus shown in Figure 8A is a single, highly distorted nucleus with extremely bright SUN staining signals and no evidence of a band-like structure. Later in prophase (Figures 8E–H), the SUN staining pattern appears to be detached (arrows, Figure 8F) from the nuclear periphery in unusual structures. The nucleus (Figures 8E–H) is called late prophase, but may instead be in between meiosis I and II. These brightly stained, small structures just outside of the *dv1* nuclei may result from the membrane vesicularization and distortion of the NE as previously reported from TEM images of *dv1* meiotic nuclei (Shamina et al., 2000). The summary observations presented here are based on visual inspection at least 50 nuclei, and 3D data collection and analysis of at least 15–20 nuclei for each of the three meiotic mutants.

From the analysis of these three classical meiosis-specific mutants of maize, we conclude that the meiotic SUN belt is tightly coupled to processes inside the nucleus. Its dynamic reorganization at zygotene, and colocalization with the telomere bouquet suggests a direct role in homologous chromosome pairing, synapsis, and recombination. Such observations further strengthen the case for the NE as a central component in the chromosome segregation pathway, conserved across multiple eukaryotic kingdoms.

## Discussion

Our findings in maize somatic tissues are in agreement with previous localization studies of SUN proteins in other plant species including tobacco, rice, and *Arabidopsis* (Van Damme et al., 2004; Moriguchi et al., 2005; Graumann et al., 2010; Oda and Fukuda, 2011). In contrast to many of these other studies that use SUN-fluorescent fusions, this report is based on 3D localization of endogenous C-terminal SUN proteins using affinity-purified peptide antibodies. The pattern of the meiotic SUN belt staining has not been previously described, to our knowledge. It is also unlike that in mouse, where patches of SUN staining colocalized to NE-associated telomere signals (Dellaporta et al., 1983; Ding et al., 2007). Indeed, the SUN belt, or anything forming a banded structure around a spherical nucleus is hitherto unreported in plants, animals, or yeast (Hasan et al., 2006; Ding et al., 2007; Penkner et al., 2007; Schmitt et al., 2007). This raises the question of whether the SUN belt described in this study represents a bona-fide meiotic LINC complex or a non-LINC structure that may still function in chromosome pairing and segregation in maize. LINC complexes are defined by SUN-domain proteins and KASH-domain proteins that connect the nucleoskeleton to the cytoskeleton across the NE. These findings predict the presence of maize KASH domain proteins in the meiotic NE, yet their identity and relationship to other plant SUN-interacting proteins remains unknown (Zhou et al., 2012, 2014; Tamura et al., 2013; Zhou and Meier, 2013).

The novel meiotic SUN belt structure described in this study may reflect a plant-specific arrangement, related to NE-associated microtubule arrays, such as the perinuclear rings described previously for plant meiosis (Shamina, 2005). Alternatively, the meiotic SUN belt may represent a more widely conserved structure in eukaryotes that has been revealed here by a combination of 3D imaging and use of antisera to stain native CCSD SUN proteins in somatic and meiotic nuclei. We suggest that this structure may represent a plant variation of the cellular machinery used to accomplish meiotic telomere movements, analogous to those associated with the horsetail chromosomal movement stage in *S. pombe* meiosis (Chikashige and Hiraoka, 2001; Chikashige et al., 2006), in *S. cerevisieae* meiosis (Conrad et al., 2008; Lee et al., 2012), or the telomere-like pairing center movements seen at meiotic prophase in *C. elegans* (Wynne et al., 2012).

These observations clearly establish a spatial coupling between the telomere bouquet and SUN2 proteins in maize, the first evidence in any plant species. They also reveal that the meiotic SUN belt mostly exists independent of the telomeres, and is not therefore likely to be formed by the telomeres themselves, but rather serve as an attachment platform for motility within a limited zone on the NE. These observations raise interesting questions about the possible need for fluidity of the SUN proteins in the belt, and the presumed SUN proteins tethered to the telomeres. Budding yeast genetic studies show that rapid chromosome movement during meiotic prophase requires a functional telomere-SUN-microtubule connection, and that these movements directly correlate with pairing interactions (Conrad et al., 2007, 2008; Lee et al., 2012). Rapid chromosome movements, including telomeric segments, and nuclear rotations have been reported in live maize meiotic cells (Sheehan and Pawlowski, 2009). Those movements may require the meiotic SUN belt, despite the lack of corresponding conspicuous actin or tubulin belt-like patterns.

Of the three meiosis-specific mutants investigated, all showed some disruption of the meiotic SUN belt, with *dy1* (Figure 6) and *dv1* (Figure 7) showing the most significant disruption. The genetic perturbation of the SUN belt by multiple different meiotic chromosome segregation mutants is consistent with our hypothesis that SUN proteins are essential for normal meiotic nuclear architecture and intra-nuclear processes including crossover control. The disruption of SUN in *dy1* (Figure 6) may not be surprising given the recent study showing how dy results in nuclear distortion and telomere-NE detachment (Murphy and Bass, 2012).

A striking result from among the mutants surveyed was the alteration of SUN staining by *dv1* (Figure 8). The *dv1* mutant has been previously reported to be defective in microtubule transition from prophase to metaphase spindle fibers (Staiger and Cande, 1990). Interestingly, *dv1* has also been shown to be defective in NE breakdown, preventing the spindle fibers from orienting in a bipolar fashion (Shamina et al., 2000). Our results reveal an even earlier phenotypic defect for *dv1*, further supporting the centrality of the meiotic NE in chromosome behavior through meiotic prophase progression.

The spatial and temporal patterns of telomeres and SUN proteins described here are summarized in Figure 9. At the leptotene stage when the telomeres are undergoing a spatial relocalization from the interior of the nucleus to the periphery, a weakly stained SUN belt is evident. At zygotene, when the telomere cluster bouquet formation is most pronounced, the telomeres are localized within the region of the retracted SUN belt. At pachytene, the full SUN ring is restored as telomeres disperse along the NE. Later in prophase, after most telomeres have detached from the NE, the meiotic SUN belt remains intact, until the NE breaks down during mitosis. Given our findings along with those from other published reports, we propose a functional model in which the maize C-terminal SUN2 protein is part of a structure that provides for telomere movement back and forth during the bouquet stage. Additional possible functions for the maize SUN belt are listed (Figure 9B) below the summary diagram to indicate that the SUN belt may serve multiple different functions, including organelle migration (arrows in zygotene stage, Figure 9A). Other likely functions to be tested in the future include promotion of homologous chromosome interactions, synapsis, resolution of homologous and non-homologous chromosome interlocks, and cross-over control.

### Conflict of interest statement

The authors declare that the research was conducted in the absence of any commercial or financial relationships that could be construed as a potential conflict of interest.

## Supplementary material

Supplemental Table 1 **Movie files for 3D deconvolution images meiotic nuclei**. The files are available here: http://4720e7f6ff230e9dea69-26c7784cfecf97d7363aa8ad6c049433.r19.cf2.rackcdn.com/Movie%20files%20for%203D%20deconvolution%20images%20meiotic%20nuclei.zip
