# Supplementary material for: A dynamic meiotic SUN belt includes the zygotene-stage telomere bouquet and is disrupted in chromosome segregation mutants of maize (Zea mays L.)
Source: Front Plant Sci. 2014 Jul 11;5:314. doi: 10.3389/fpls.2014.00314 (PMC4093829; doi:10.3389/fpls.2014.00314)
Supplement: Supplemental Table 1 — Movie files for 3D deconvolution images meiotic nuclei. The files are available here: http://4720e7f6ff230e9dea69-26c7784cfecf97d7363aa8ad6c049433.r19.cf2.rackcdn.com/Movie%20files%20for%203D%20deconvolution%20images%20meiotic%20nuclei.zip [file DataSheet1.DOCX]

**Supplemental Table 1. Movie files for 3D deconvolution images meiotic nuclei. The files are available here:** <http://4720e7f6ff230e9dea69-26c7784cfecf97d7363aa8ad6c049433.r19.cf2.rackcdn.com/Movie%20files%20for%203D%20deconvolution%20images%20meiotic%20nuclei.zip>

| **3D DataSet ID^a^** | **Genotype^b^**  **Substage^c^**  **Wavelength^d^**  **(staining target)** | **Names^e^ of individual movie (.mov) files** |
| --- | --- | --- |
| 01_LEPTO_ZmSUN2  (6 movies) | WT  LEPTOTENE  FITC (Immuno-SUN2) | 1a_LEPTO_Imm262c_DAPI  1b_LEPTO_Imm262c_DAPI_spin360  1c_LEPTO_Imm262c_FITCsun2  1d_LEPTO_Imm262c_FITCsun2_spin360  1e_LEPTO_Imm262c_DAPIred-FITCgreen  1f_LEPTO_Imm262c_DF_spin360 |
| 02_ZYGO_ZmSUN2  (6 movies) | WT  ZYGOTENE  FITC (immuno-SUN2) | 2a_ZYGO_Imm262s_DAPI  2b_ZYGO_Imm262s_DAPI_spin360  2c_ZYGO_Imm262s_FITCsun2  2d_ZYGO_Imm262s_FITCsun2_spin360  2e_ZYGO_Imm262s_DAPIred-FITCgreen  2f_ZYGO_Imm262s_DF_spin360 |
| 03_PACHY1_ZmSUN2  (6 movies) | WT  PACHYTENE (Ex. 1)  FITC (immuno-SUN2) | 3a_PACHY1_Imm371y_DAPI  3b_PACHY1_Imm371y_DAPI_spin360  3c_PACHY1_Imm371y_FITCsun2  3d_PACHY1_Imm371y_FITCsun2_spin360  3e_PACHY1_Imm371y_DAPIred-FITCgreen  3f_PACHY1_Imm371y_DF_spin360 |
| 04_PACHY2_ZmSUN2  (6 movies) | WT  PACHYTENE (Ex. 2)  FITC (immuno-SUN2) | 4a_PACHY2_Imm261g_DAPI  4b_PACHY2_Imm261g_DAPI_spin360  4c_PACHY2_Imm261g_FITCsun2  4d_PACHY2_Imm261g_FITCsun2_spin360  4e_PACHY2_Imm261g_DAPIred-FITCgreen  4f_PACHY2_Imm261g_DF_spin360 |
| 05_ZYGO1_ZmSUN2  (8 movies) | WT  ZYGOTENE (Ex. 1)  FITC (Telomere FISH); RHOD (immuno-SUN2) | 5a_ZYGO1_ImmFish34k_DAPI  5b_ZYGO1_ImmFish34k_DAPI_spin360  5c_ZYGO1_ImmFish34k_FITCtelo  5d_ZYGO1_ImmFish34k_FITCtelo_spin360  5e_ZYGO1_ImmFish34k_RHODsun2  5f_ZYGO1_ImmFish34k_RHODsun2_spin360  5g_ZYGO1_ImmFish34k_Dblue-Fgreen-Rred  5h_ZYGO1_ImmFish34k_DFR_spin360 |
| 06_ZYGO2_ZmSUN2Telo  (8 movies) | WT  ZYGOTENE (Ex. 2)  FITC (Telomere FISH); RHOD (immuno-SUN2) | 6a_ZYG02_ImmFish51e_DAPI  6b_ZYG02_ImmFish51e_DAPI_spin360  6c_ZYG02_ImmFish51e_FITCtelo  6d_ZYG02_ImmFish51e_FITCtelo_spin360  6e_ZYG02_ImmFish51e_RHODsun2  6f_ZYG02_ImmFish51e_RHOD_spin360  6g_ZYG02_ImmFish51e_Dblue-Fgreen-Rred  6h_ZYG02_ImmFish51e_DFR_spin360 |
| 07_ZYGO_ZmTubulin  (6 movies) | WT  ZYGOTENE  FITC (immuno-tubulin) | 7a_ZYGO_ImmGC8m_DAPI  7b_ZYGO_ImmGC8m_DAPI_spin360  7c_ZYGO_ImmGC8m_FITCtub  7d_ZYGO_ImmGC8m_FITCtub_spin360  7e_ZYGO_ImmGC8m_DAPIred-FITCgreen  7f_ZYGO_ImmGC8m_DF_spin360 |
| 08_PACHY_ZmActin  (6 movies) | WT  ZYGOTENE/PACHY  RHOD (F-Actin, Rhodamine phalloidin) | 8a_PACHY_ta1rp7_DAPI  8b_PACHY_ta1rp7_DAPI_spin360  8c_PACHY_ta1rp7_RHODactin  8d_PACHY_ta1rp7_RHODactin_spin360  8e_PACHY_ta1rp7_DAPIred-RHODgreen  8f_PACHY_ta1rp7_DR_spin360 |
| 09_PACHYdy_ZmSUN2  (6 movies) | *desynaptic1* (*dy1*)  PACHYTENE  FITC (immuno-SUN2) | 9a_PACHYdy_Imm311b_DAPI  9b_PACHYdy_Imm311b_DAPI_spin360  9c_PACHYdy_Imm311b_FITCsun2  9d_PACHYdy_Imm311b_FITCsun2_spin360  9d_PACHYdy_Imm311b_DAPIred-FITCgreen  9e_PACHYdy_Imm311b_DF_spin360 |
| 10_ZYGOas_ZmSUN2  (6 movies) | *asynaptic1* (*as1*)  ZYGOTENE  FITC (immuno-SUN2) | 10a_ZYGOas_Imm363k_DAPI  10b_ZYGOas_Imm363k_DAPI_spin360  10c_ZYGOas_Imm363k_FITC  10d_ZYGOas_Imm363k_FITC_spin360  10e_ZYGOas_Imm363k_DAPIred-FITCgreen  10f_ZYGOas_Imm363k_DF_spin360 |

Footnotes:

^a^ Image datasets (10 total) converted to QuickTime movie (.mov) files to show step-through optical Z-sections or Y-axis spinning projections. For each movie type, data are shown as single-wavelength grey-scale movies or multiple-wavelength color movie files.

^b^ WT indicates normal (similar to wild-type) genotype; homozygous meiosis-specific mutant alleles of *desynaptic* (*dy1/dy1*) or *asynaptic* (*as1/as1*) maize.

^c^ cell type; substage of prophase I of male meiosis, staging based on criteria from DAPI-stained chromatin morphology as described by Bass *et al*., (1997).

^d^ FITC, FITC channel; RHOD, rhodamine channel; (not listed, DAPI, used to counterstain total chromatin in all datasets). Target molecule being localized/stained is indicated in parentheses.

^e^ Movie File names indicate DataSet ID (01-10); individual movies (a-f or a-h), stage; experimental reference ID (“Imm” for immunofluorescence, “Fish” for FISH), wavelength (DAPI/D, FITC/F, rhodamine/RHOD/R); and “spin360” for spinning projections. Multiple wavelength files are pseudo-colored differently to maximize visibility of image data and to emphasize one stain by display as green. Two-wavelength images are shown with red for DAPI and green for FITC (datasets 01-04, 08-10) or green for Rhodamine (dataset 07). Three-wavelength images (datasets 05, 06) are shown with blue for DAPI, green for FITC, and red for RHOD. All movies show Step-Through Sections of the entire 3D subset of data, one optical section at a time, except for the spinning projections (“_spin360”), which show through-focus maximum-intensity projections viewed at progressive angles about the Y-axis. File section numbers and scale bars are displayed in the step-through projections.
